# Extraction, Purification, Characterization, and Wound Healing Effects of Novel Prickly Pear (*Opuntiaficus-indica* (L.) Mill.) Heteropolysaccharides

**DOI:** 10.3390/ph17101410

**Published:** 2024-10-21

**Authors:** Naourez Ktari, Wafa Gargouri, Lobna Jlaiel, Imen Trabelsi, Sirine Ben Slima, Sana Bardaa, Farida Bendali, Riadh Ben Salah

**Affiliations:** 1 Laboratory of Biotechnology Microbial, Enzymatic and Biomolecules, Centre of Biotechnology of Sfax, Road of Sidi Mansour Km 6, P.O. Box 1177, Sfax 3018, Tunisia; naourez.ktari@yahoo.fr (N.K.); gargouri.wafa@yahoo.fr (W.G.); imentrabelsi@live.fr (I.T.); benslimasirine@yahoo.fr (S.B.S.); sanabardaa@gmail.com (S.B.); 2 Department of Life Sciences, Faculty of Science of Gabes, Omar Ibn Khattab Street, Gabes 6029, Tunisia; 3 Analytical Service, Centre of Biotechnology of Sfax, Road of Sidi Mansour Km 6, P.O. Box 1177, Sfax 3018, Tunisia; lobna222@gmail.com; 4 Laboratoire de Microbiologie Appliquée, Faculté des Sciences de la Nature et de la Vie, Université de Bejaia, Bejaia 06000, Algeria; farida.bendali@univ-bejaia.dz

**Keywords:** prickly pear, monosaccharide composition, structural characteristics, antioxidant and antibacterial activity, HEK-293 cells

## Abstract

**Background:** The present study undertakes the purification of a novel polysaccharide from Tunisian prickly pear *(Opuntiaficus-indica* (L.) Mill.) rackets (PPPRs) and the determination of its physicochemical properties, structure, antibacterial and antioxidant properties, as well as its in vitro and in vivo wound healing potential. **Methods:** The PPPR was structurally analyzed by Fourier Transform Infrared Spectroscopy (FTIR) and UV/Visible Spectroscopy, revealing characteristic bands of polysaccharides. According to thin-layer chromatography (TLC), high-performance liquid chromatography (HPLC), and Gas Chromatography–Mass Spectrometry (GC–MS) analyses. **Results:** The crude PPPR is an heteropolysaccharide composed of glucose (62.4%), galactose (19.37%), mannose (10.24%), and rhamnose (7.98%), with an average molecular weight of 90.94 kDa. This novel polysaccharide exhibited notable antioxidant potential assessed by four different in vitro assays: the 2-diphenyl-1-picrylhydrazyl (DPPH) scavenging assay, ferric reducing power, ferrous chelating activity, and scavenging activity against 2,2′-azino-bis-3-ethylbenzothiazoline-6-sulphonic acid (ABTS). In addition, the PPPR displayed high antibacterial activities with a MIC of 2.5 mg/mL against *Salmonella Typhimurium* and *Pseudomonas aeruginosa*, cytocompatibility properties, and non-cytotoxicity. Subsequently, the effect of the PPPR on skin wound healing was studied in a diabetic rat model induced by alloxan, revealing a significant acceleration in the wound healing process. This acceleration was evidenced by the expedited recovery of the dermis, increased formation of blood vessels, and enhanced tissue granulation. **Conclusion:** Therefore, the findings offer fresh perspectives on the creation of a potentially efficient and promising racket polysaccharide-based therapy for the treatment of persistent diabetic wounds.

## 1. Introduction

Diabetes is a widespread disorder characterized by disruptions in the endocrine and metabolic systems, which often results in prolonged and impaired wound healing. This delay in the healing process can have a profound and negative effect on the overall quality of life for those affected, leading to additional health challenges and reduced well-being [1]. Changes to the vascular basement membrane in tissues decrease collagen expression, and alterations in immune responses contribute considerably to the exacerbation of pain and complications in wound healing. Furthermore, excessive glycation gives up merchandise, and hyperglycemia may cause damage to peripheral blood micro-vessels and larger vessels, while also inhibiting the expression of various neurotrophic and vascular factors [2]. Several physiological elements are decreased, including the proliferation of fibroblasts and keratinocytes, angiogenesis, and collagen deposition, which can be related to ineffective treatment outcomes for diabetic wounds. Hence, it is very pressing to discover a new healing treatment for diabetic wound restoration [3].

A hydrogel is a polymer biomaterial that supplies purposeful molecules, drugs, and cells in diverse therapeutics for its biological, biodegradable, and biocompatible characteristics [4]. Indeed, hydrogels are used in a wide range of applications, namely as adsorbents for removing pollutants in aqueous environments [5]. In addition, hydrogels are known for their ability to protect damaged areas from external stimuli and maintain a moist environment, which is conducive to wound healing. Indeed, hydrogel-based treatments have shown promise in various medical applications, including wound care and drug delivery systems [4,6].

Plant polysaccharides are useful in the development of new medicines, functional foods, and nutrients. In fact, these biopolymers present several biological effects, such as antioxidant, immunomodulatory, antibacterial, antiviral, hypoglycemic, hypolipidemic, antitumor, antiradiation, and hepatoprotective functions [7]. Scientists in medicine, agronomy, and food chemistry are focused on cacti because of their long history of medicinal use, minimal cultivation needs, and potential as a basis for developing a green vegetable. Cactus is the generic name of a species in the family Cacti, which consists of approximately 127 genera and 1750 known species [3]. Recently, polysaccharides are considered the most important active component of cactus and have become a main topic in *O. ficus-indica* research. Cactus polysaccharides include heteropolysaccharides, homopolysaccharides, and glycoproteins with various polysaccharide concentrations, monosaccharide composition ratios, and relative molecular weights [8]. Polysaccharides extracted from *O. ficus-indica* appear to have a few positive impacts on skin-repairing properties and are anti-inflammatory and chondroprotective [8]. These biopolymers, other than the high molecular weight components, contain metabolites with distinctive chemical natures and atomic weight dissemination. The polysaccharides were utilized in a wound healing test to assess their cicatrizing ability, which suggested faster dermal recovery.

The present research focuses on the extraction, purification, and characterization of a novel polysaccharide from Prickly pear rackets (PPPRs) and, for the first time, explores its effect on chronic wound healing in diabetic rat models.

## 2. Results and Discussion

### 2.1. Biochemical and Color Analysis of PPPRs

The use of polysaccharides is primarily dependent on their physicochemical characteristics. Table 1 provides a summary of the findings from the biochemical study of PPPR. Various factors, such as extraction duration, temperature, and water/raw material ratio, can significantly affect polysaccharide yield during the extraction process. Nevertheless, the current study’s findings are consistent with those of several other studies [9,10]. As a result, the extraction yield of PPPRs was 23.17*%*. Yilmaz et al. [11] optimized the extraction of polysaccharides from *O. ficus-indica* and found that the maximum yield was 18.58%. The differences in extraction yields may be attributed to various factors, including environmental conditions, physiological factors, habitat, growth cycle, and seasonal fluctuations [12]. Moreover, results from the phenol-sulfuric acid test revealed that the carbohydrate content of the extracted polysaccharide was about 91.02%. The high carbohydrate content observed was comparable to those reported by Cheikh Rouhou et al. [13], who reported that the carbohydrate content of the polysaccharide extracted from cactus rackets (*O. ficus-indica*) was 83.22%. The percentages of moisture and ash were 5.11*%* and 2.69%, respectively. The analysis indicated that the PPPR was free of fat and contained a relatively low fraction of protein (1.1%). Color is an appealing characteristic that can define the influence of the PPPR in the perceived quality of food products. The lightness value L*, which ranges from 0 (black) to 100 (white), is equivalent to L* = 92.44 ± 0.05. The polysaccharide’s dark color is described by the values of a* and b*, which were recorded at 0.17 ± 0.00 and 2.23 ± 0.11, respectively, being attributed to the light yellow color of the PPPR.

### 2.2. Analysis of the PPPR

#### 2.2.1. Ultraviolet Light Absorption Data

**Figure 1:**
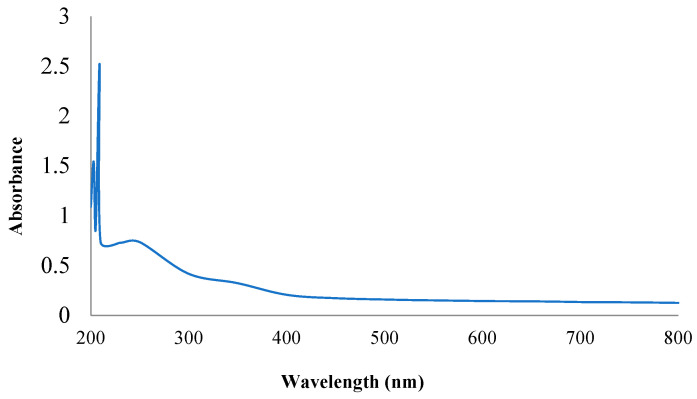
Scan of the PPPR within the wavelength range of 200–800 nm.

The maximum absorption peaks of the PPPR were recorded in the UV/visible spectrum, ranging from 200 to 800 nm (Figure 1). Based on their absorption maxima between 204–230 nm and the absence of any absorption within the 260–280 nm range, it was determined that the PPPR was identified as a polysaccharide rather than a protein or nucleic acid [14].

#### 2.2.2. FTIR Spectroscopy Measurements

**Figure 2:**
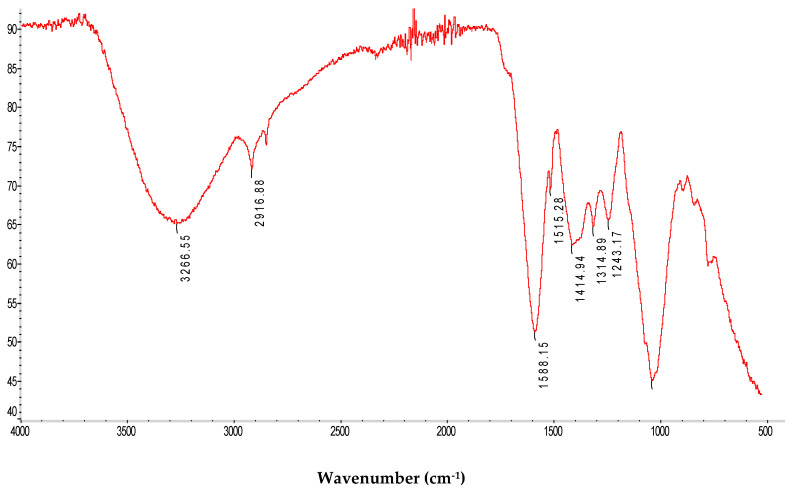
Fourier transform infrared spectrum of the PPPR.

FTIR was conducted to identify the bonds and the functional groups present in the extracted polymers. The obtained spectra were analyzed by identifying the peaks corresponding to a characteristic pattern or a specific bond. The FTIR spectra of the purified polysaccharide are shown in Figure 2. As demonstrated, typical peaks of the polysaccharide are present at 3266.55, 2916.88, 1588.15, 1515.28, 1414.94, 1314.89, and 1243.17 cm^−1^. The presence of a broad and intense band between 3200–3500 cm^−1^ is assigned to the stretching vibration of the hydroxyl groups (-OH), which is characteristic of polysaccharides, as well as to the absorption of water [15]. The intense band at 3266.55 cm^−1^ corresponds to the elongation vibration of OH groups due to inter- and intra-molecular hydrogen bands [16]. Weak bands corresponding to the asymmetric vibrations of the C-H bonds were observed between 2800 and 3000 cm^−1^. The peak observed at 2916.88 cm^−1^ represented the stretching of the C-H groups of the free sugars [17]. Furthermore, the absorption noticed at 1588.15 cm^−1^ determines the existence of the carboxylate CO bonds within the acylamino group [16]. According to the previously elaborated literature [18], the most significant peaks are those detected at 1314 cm^−1^ and 1230 cm^−1^, generated from the bending vibration of the stretching vibration of the sulfate ester groups (S=O). The most significant peaks refer to those observed at 1414.94 cm^−1^ and 1314.89 cm^−1^ and are attributed to the presence of the sulfate ester groups (S=O). Finally, the band at 1243.17 cm^−1^ highlights the presence of acetyl groups from pectic residues as reported in the literature [19].

#### 2.2.3. Monosaccharide Composition Analysis

- *TLC analysis and HPLC spectrum*

**Figure 3:**
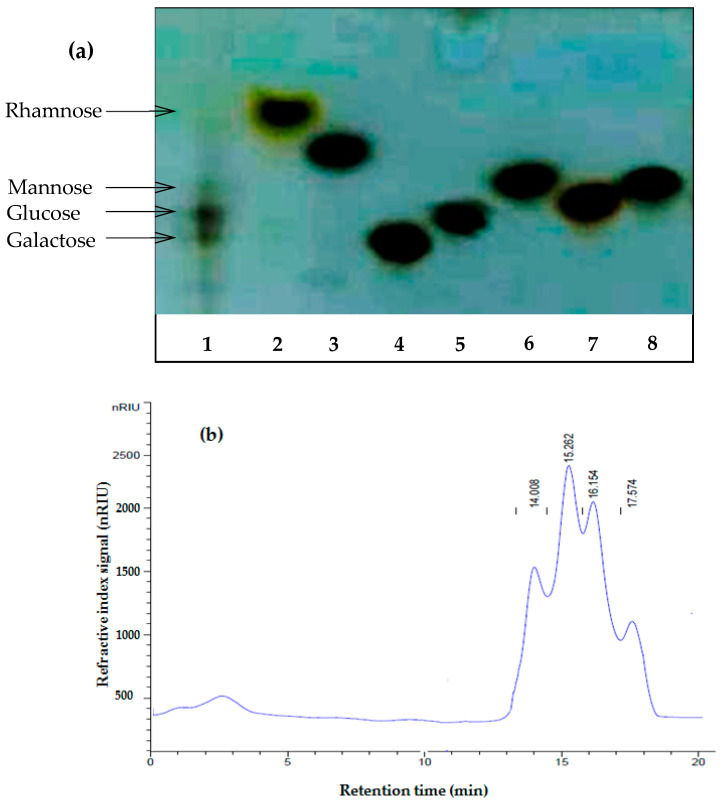
Monosaccharide composition of the PPPR. (**a**) TLC of the PPPR, (1) hydrolyzed PPPR. (2) Rhamnose, (3) xylose, (4) galactose, (5) glucose, (6) arabinose, (7) mannose, and (8) fructose were used as standards. (**b**) HPLC chromatogram profiles of PPPR.

The monosaccharide composition of the PPPR determination was conducted by TLC analysis (Figure 3a). The findings reveal the existence of four plugs with retention factors of 0.74 (rhamnose), 0.58 (glucose), 0.56 (galactose), and 0.61 (mannose).

To confirm the obtained results, the PPPR was explored through HPLC analysis. The chromatograms revealed the presence of four peaks at 14.008, 15.262, 16.154, and 17.574 (Figure 3b), corresponding to those of glucose, mannose, galactose, and rhamnose, respectively, which were used as standards. These results confirm that the PPPR is a heteropolysaccharide consisting of a chain of glucose, mannose, galactose, and rhamnose with weighs of 23.55, 23.89, 23.78, and 23.47 mg/100 mg of PPPR, respectively.

- *Gas chromatography–mass spectrometry (GC–MS) analysis*

The monosaccharide composition of the PPPR was determined by using GC–MS to analyze their trimethylsilyl (TMS) residues. The identification of the peaks was carried out by comparing retention times and mass spectra with those obtained by injections of pure standards. Monosaccharide composition is presented in Table 2. The obtained results revealed the presence of different carbohydrate moieties in varied proportions (Table 2). Acid hydrolysis of the PPPR showed that glucose was the most abundant sugar (62.4%), followed by galactose (19.37%), mannose (10.24%), and rhamnose (7.98%). These results confirm HPLC analysis findings.

#### 2.2.4. Molecular Weight Determination

**Figure 4:**
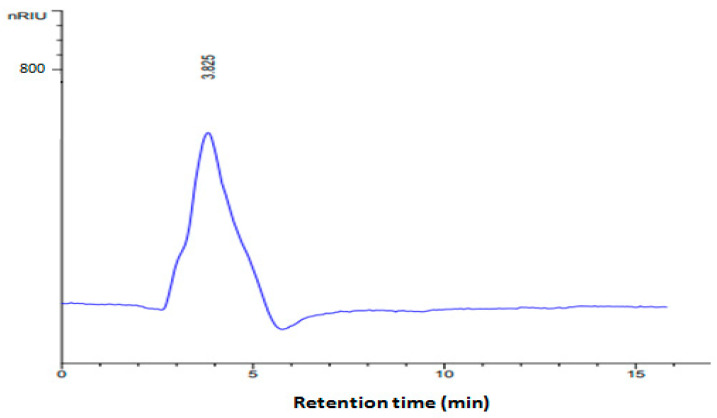
Gel filtration chromatographs of the PPPR.

The average molecular weight (Figure 4) of the PPPR exhibited a major peak at a retention time of 3.825, corresponding to an approximate value of 90.94 kDa. The origin of polysaccharides significantly influences their average molecular weight. For instance, Pawar and coauthor [20] found that the average molecular weight of the purified polysaccharide from *Senna tora* (L.) seeds was 198 kDa. Similarly, Wang et al. [21] demonstrated that various fractions extracted from *Camellia Sinensis* (L.) (tea seeds) constituted homogeneous polysaccharides with average molecular weights of 4.588 kDa, 500 kDa, and 100 kDa.

#### 2.2.5. Differential Scanning Calorimetry (DSC)Analysis

**Figure 5:**
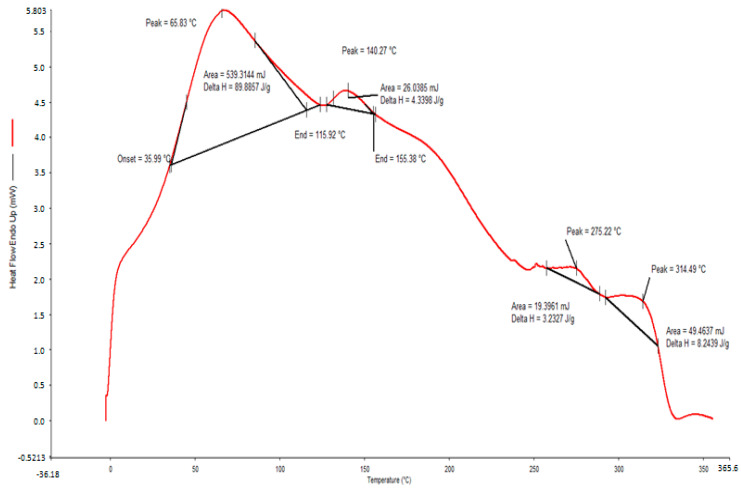
Differential scanning calorimetry results for the thermal behavior of the PPPR.

The transition temperature and thermal behavior of polysaccharides determine their structural, functional, and physiochemical properties [22]. The thermal behavior of polysaccharides extracted from *O. ficus indica* was studied by DSC analysis. The DSC analysis of purified polysaccharides revealed various endothermic and exothermic peaks (Figure 5). The glass transition temperature (Tg) of the PPPR was exhibited by a peak at 65.83 °C. The exothermic event was observed at 140.27, 275.22, and 314.49 °C, which were considered to represent the melting temperatures of the extracted polysaccharides. Previous studies have shown that the melting temperature of polysaccharides extracted from psyllium leaves using various extraction methods ranges from 240 to 350 °C [22]. Furthermore, the varied thermal transitions depend upon both the temperature conditions and the chemical structure of the polymer. The transition temperature of polysaccharides is influenced by the specific composition of monosaccharides, and it rises in tandem with an increase in the polymerization degree [23].

### 2.3. Biocompatibility Analysis

**Figure 6:**
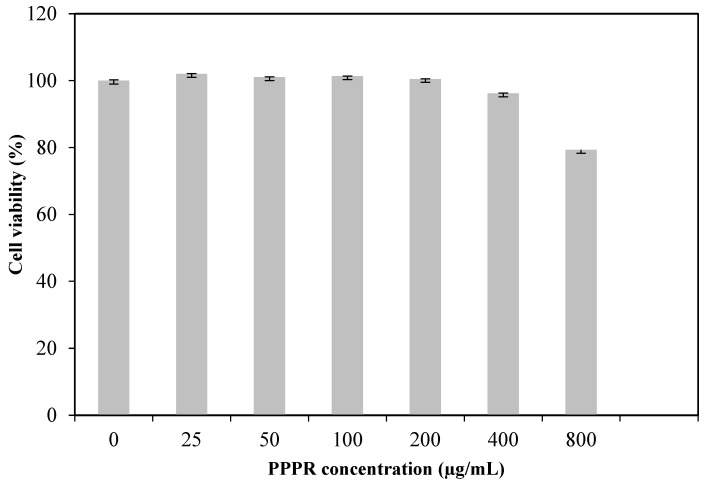
Effects of the PPPR on HEK-293 cell viability.

The safety of the PPPR was assessed using normal HEK-293 cells cultured with several doses of polysaccharide, ranging from 0 to 800 μg/mL. The MTT test was utilized to identify cell viability. The results presented in Figure 6 demonstrate that co-culturing with the PPPR for 48 h did not significantly lower cell viability, which remained at over 80% at concentrations ranging from 25 to 800 μg/mL, as compared to the cell viability of the control group. The results clearly showed that the PPPR, when administered at doses of up to 0.8 mg/mL, displayed excellent compatibility with cells and was confirmed to be non-toxic to human cells. Polysaccharides from plant materials are widely recognized in the scientific literature for their non-toxic and biocompatible nature as a biopolymer [24]. Furthermore, Amaral et al. [25] reported that two polysaccharide fractions extracted from the pulp of gabiroba showed cytotoxic effects against human glioblastoma cancer cells, even at low concentrations, without showing any observed cytotoxicity in normal fibroblast cells.

### 2.4. Antibacterial Potential of the PPPR In Vitro

According to the disk diffusion method, the PPPR exhibited varying degrees of inhibition against two Gram-negative bacterial strains (*Salmonella Typhimurium* and *Pseudomonas aeruginosa)* at a concentration of 10 mg/mL. The inhibitory zones for these strains measured between 6 and 7 mm, respectively. In the present research, the PPPR exhibited notable antimicrobial potential against *S. Typhimurium* and *P. aeruginosa*. The MIC for the PPPR was 2.5 mg/mL for both tested pathogenic bacteria. The antibacterial activity inhibition concentration (IC50) of the PPPR was evaluated at 30 mg/mL against *S. Typhimurium* and 32 mg/mL for *P. aeruginosa*. The PPPR was indicated to be a significant and active constituent for food and pharmaceutical businesses. Consistent with prior findings [26], it was observed that polysaccharides extracted from *Chaetomium globosum* exhibited higher antimicrobial activity against *S. aureus* compared to *E. coli*. Similarly, another study indicated that the antimicrobial activity of polysaccharides derived from the fruits of *Broussonetia papyrifera* (L.) against *S. aureus* surpassed that against *E. coli* [27].

### 2.5. Antioxidant Potential of the PPPR In Vitro

#### 2.5.1. DPPH Assay

**Figure 7:**
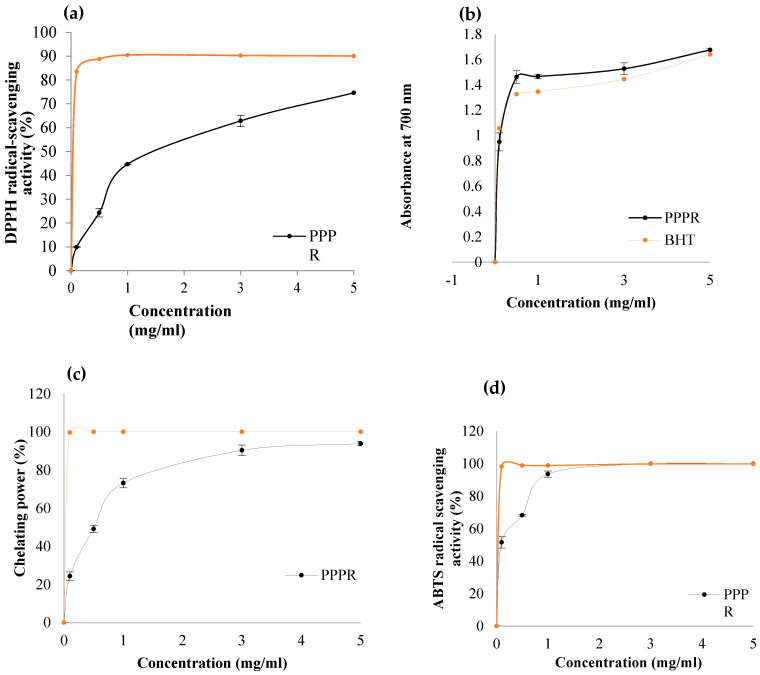
Antioxidant activities of the PPPR at different concentrations.(**a**) Scavenging effect on the DPPH free radical, (**b**) reducing power, (**c**) metal chelating activity, and (**d**) ABTS scavenging activity. BHT, EDTA, and Trolox were used as positive controls. All analyses were carried out in triplicate.

DPPH, functioning as a stable free radical, is commonly employed to assess the ability of natural compounds to scavenge free radicals by providing hydrogen and forming a stable DPPH molecule [28]. The free radical scavenging capability of the PPPR was assessed and compared with the synthetic antioxidant BHT. Figure 7a represents the DPPH scavenging capacity of the PPPR at varying concentrations (1–5 mg/mL), showing that the DPPH radical scavenging activity of the PPPR increased with the concentration of polysaccharide, reaching a maximum of 75% at a concentration of 5 mg/mL.The PPPR presented an antioxidant activity on the DPPH radical, with an IC50 of 2.6 mg/mL.A previous study shows that the IC50 from a polysaccharide from *Sorghum bicolor* L. was 8.5 mg/mL [23]. The notable radical scavenging activity of the PPPR is likely due to the hydroxyl and carboxyl groups present in the polysaccharides. These groups act as hydrogen donors, allowing them to scavenge DPPH free radicals and subsequently mitigate the effects of oxidative stress. Consequently, the obtained results suggest that the PPPR could serve as a promising natural source of antioxidant agents.

#### 2.5.2. Ferric Reducing Power

The ability of the PPPR to reduce Fe^3+^ to Fe^2+^ was assessed by measuring the absorbance at 700 nm, which indicates the formation of Perl’s Prussian blue. Figure 7b illustrates that the ferric reducing power increased proportionally with the concentrations of the PPPR, ranging from 1–5 mg/mL. Nevertheless, the PPPR exhibited relatively higher activity compared to BHT. Previous reports indicate that the reducing power of plant polysaccharides is often linked to their ability to react with specific peroxide precursors [29].

#### 2.5.3. Ferrous Chelating Activity

By interfering with the formation of the Fe^2+^–ferrozine complex, chelating agents have the potential to inhibit lipid oxidation. In our investigation, a polysaccharide from *O. ficus indica* demonstrated remarkable chelating activity, escalating in a dose-dependent manner and reaching 93.95% at a concentration of 5 mg/mL. Nonetheless, this activity fell short compared to ethylenediaminetetraacetic acid (EDTA) in all tested concentrations (Figure 7c). Furthermore, the chelating activity of *O. ficus indica* polysaccharide exceeded that of laminarin isolated from *Cystoseirabarbata*, which was 78% at a concentration of 20 mg/mL [30]. The IC50 value for the Fe^2+^ ion chelating capacity of the PPPR was determined to be 1.26 mg/mL. The chelating potentialities of galacturonic acid units are greatly increased when carboxyl groups are present [31].

#### 2.5.4. ABTS^+^ Assay

ABTS^+^ was another assay employed to assess the antioxidant capacity of foods and natural products [32]. The ABTS^+^ scavenging ability of the PPPR is illustrated in Figure 7d. Scavenging activities increased with rising concentrations from 1 to 5 mg/mL. In this experiment, trolox served as a positive control in this experiment, demonstrating a dose-dependent increase in ABTS^+^ scavenging potential, reaching 100% at 5 mg/mL, with an IC50 value of BHT at 0.01 mg/mL. The ABTS^+^ scavenging activity of the PPPR was expressed as a percentage of the maximum activity reported for the positive control. At concentrations of 3 to 5 mg/mL, the PPPR exhibited 100% activity, with a corresponding IC50 value of 0.13 mg/mL. Our findings exceeded those documented by Mzoughi et al. [32], who reported an IC50 value of 1.1 mg/mL for polysaccharides extracted from quinoa.

### 2.6. In Vivo Effect of the PPPR on Diabetic Wounds in Rats

**Figure 8:**
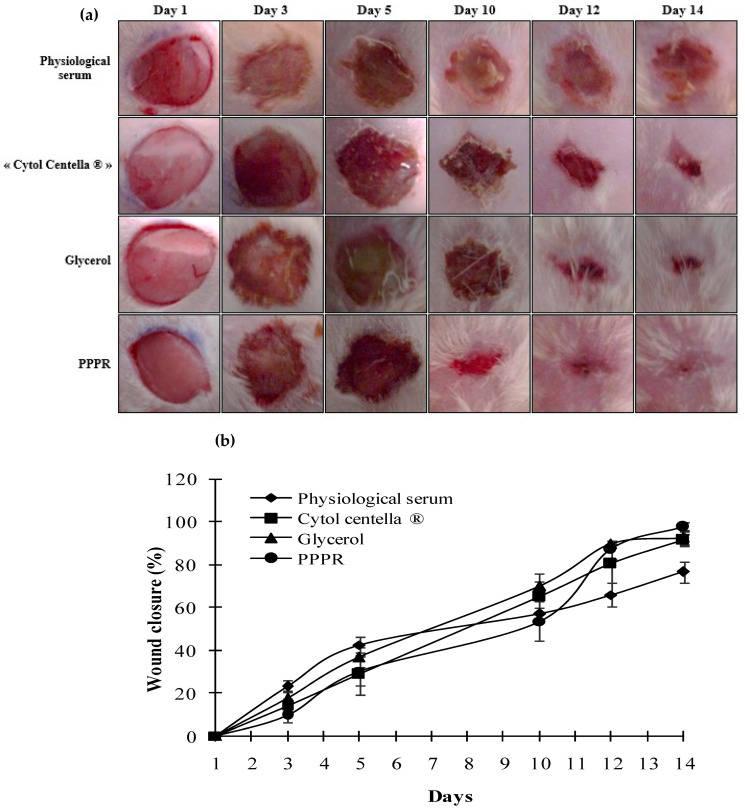
(**a**) Photographs of wounds taken of diabetic rats treated with physiological serum, Cytolcentella^®^, glycerol, or PPPR (15 mg/mL) on days 1, 3, 5, 10, 12, and 14, (**b**) Wound healing rate (%) for different treatment groups on wounds.

Alloxan is a well-recognized diabetogenic substance frequently employed in creating a model of type I diabetes mellitus for experimental diabetes research [33]. In this work, rats injected with alloxan exhibited an average blood glucose level exceeding 200 mg/dL, accompanied by polyuria and a noticeable reduction in body weight, which indicates successful induction of the diabetic model. In the present rat wound healing model, we induced a single full-thickness oval skin wound, approximately 150 mm^2^ in size, on the shaved dorsal interscapular region of each diabetic rat. The wound healing capacity of the PPPR involved examining various parameters, such as the wound closure rate, epithelialization time, inflammation status, changes in wound color, and histological analysis of the regenerated tissue. Monitoring changes in the skin wound area to assess wound closure is commonly used due to its accessibility, ease of handling, and clinical relevance. Figure 8a displays representative images of wounds from different groups of rats taken on days 1, 3, 5, 10, 12, and 14. Notably, throughout the treatment period, macroscopic examination of wounds treated with the PPPR did not reveal any related signs of infection or inflammation, such as pus, discharge, or unpleasant odor. Furthermore, there was no discernible delay in healing action. Conversely, untreated wounds in Group 1 exhibited significant delays in healing, attributed to impaired blood circulation in diabetic rats. Even by the end of the experiment on day 14, wounds in this group remained open with surrounding red tissues. Following the local application of extracted polysaccharide and Cytol Centella^®^ cream, both treated groups displayed a noticeable dark red coloration on the third day. This observation suggested the start of the healing process, marked by the development of blood clots containing cellular debris. In addition, wounds in both treated groups displayed healthy brown coloration on the 5th day. This change was attributed to plasma exudation, accompanied by the formation of superficial crusts at the wound site, which persisted until the 10th day.

However, wounds treated with the reference product, CytolCentella^®^ cream, exhibited harder, thicker, and darker crusts surrounding the wound area compared to those treated with the PPPR, resulting in a drier wound environment. Numerous studies have indicated that crusts serve as a physical barrier, safeguarding the skin wound from external infections and pathogens. However, rapid migration of epidermal cells is facilitated by a less dry wound, thereby expediting the epithelialization process [23,34]. By day 12, in the group of rats treated with the PPPR, the crust began to slough off, allowing new skin tissue to grow, leading to the emergence of a pinkish hue. Complete wound closure was observed by the 14th day in these rats. In contrast, untreated animals still displayed open wounds surrounded by red tissues upon finishing the experiment.

Figure 8 illustrates the quantified wound areas of each group at various time points. The group treated with the PPPR gel exhibited a faster progression of mean percentage contraction, reaching 100% by the 14th day, indicating complete healing. However, the group treated with “Cytol Centella^®^”, the glycerol-treated group, and the control group showed delayed wound contraction progressions, reaching 89.44%, 90.29%, and 73%, respectively, by the 14th day. Consequently, the polysaccharide extracted from *O. ficus indica* appears to have a greater wound healing potential compared to that of « Cytol Centella^®^ ». These results may be attributed to the protective effects of polysaccharides on the skin, thanks to their antioxidant and anti-aging properties. In addition, polysaccharides can regulate or limit the migration of moisture, thereby maintaining a moist environment, which helps to protect the wound from infections, contributing to a faster wound healing process.The overall findings imply that PLS significantly accelerates wound healing in diabetic rats induced by alloxan, largely owing to its role in activating the immune system and macrophages, which are crucial for wound site cleanup post-injury [35].

**Figure 9:**
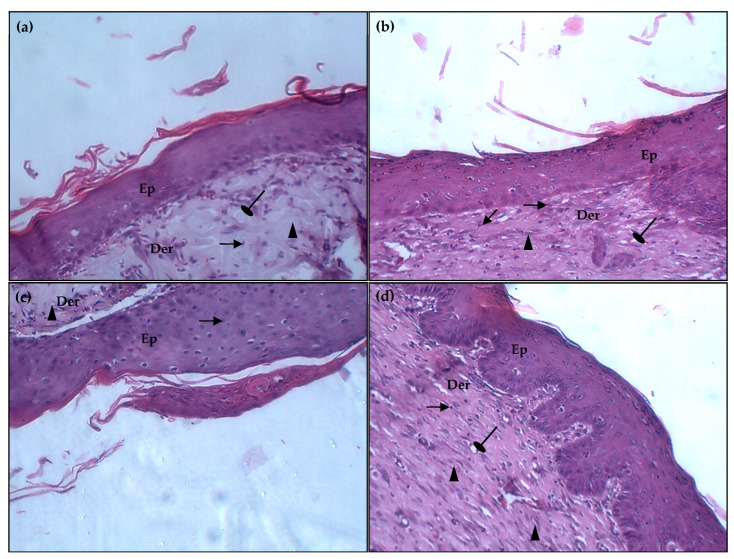
Histological hematoxylin–eosin staining analysis of wounded skin tissue sections in the diabetic rats treated with physiological serum (**a**), « Cytol Centella^®^ » (**b**), glycerol (**c**), and PPPR (**d**) on the 14th day post-wounding (×400 magnification). Der: Dermis; Ep: Epidermis; (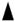): Collagen; (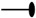): Vessels; (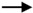): inflammatory cells.

Images taken from tissue biopsies of wound sections from each group on the 14th day after upon excision, after hematoxylin–eosin (HE) staining, showed rapid recovery of normal tissue structures following the application of the PPPR compared with the reference drug-treated group and the diabetic control group (Figure 9).

In diabetic animal wounds, there was a noticeable delay in healing, characterized by irregular and incomplete epidermal morphology, as illustrated in Figure 9. Additionally, the dermis of the« Cytol Centella^®^ » and glycerol-treated groups (Figure 9b,c) exhibited a moderate infiltration of inflammatory cells, indicating a chronic stage of inflammation, along with pronounced hyperemia of capillary blood vessels. Furthermore, it is noteworthy to observe a foreign body reaction, which is considered likely to be the consequence of gauze fiber adhering to the healing tissue and subsequent penetration into the dermis of the wound tissue after being surrounded by newly grown tissue. The granulation tissue exhibited an accumulation of macrophages and a moderate presence of collagen fibers. The hematoxylin–eosin staining of biopsies from rats treated with the PPPR revealed complete epidermal regeneration with well-structured layers covering the entire wound surface (Figure 9d). These biopsies exhibited a high density of fibroblasts and collagen, along with some macrophages. Additionally, they demonstrated significant neovascularization and complete re-epithelialization, characterized by a well-differentiated epidermis. Collagen deposits appeared more prominently than in other groups, forming a broad, organized, and compact fibrous zone. Furthermore, a low relative volume of inflammatory cells was observed, suggesting an anti-inflammatory effect of this tested healing agent. In fact, complete re-epithelization was visible, complete with a well-organized layer of epidermis, quicker keratinization, increased collagen density, and freshly created blood vessels populating the dermal. According to the chromatic study, all these characteristics showed that the wound-healing effect of the PPPR was superior to that of the well-known healer, Cytol Centella^®^ cream. Numerous factors have been documented to contribute to promoting the wound healing process, including antioxidant properties, mitogenic effects, and moisturizing abilities [36].

Antioxidants have proven effective in improving the wound microenvironment by neutralizing excessive reactive oxygen species, which in turn reduces oxidative stress and accelerates the healing process. Moreover, exhibiting antibacterial and biofilm-preventing activities is considered to be a viable option for treating chronic wounds.

It is noteworthy that the antioxidant properties and antibacterial activities of the PPPR could be considered the most effective contributing factors to enhancing diabetic wound healing. The utilization of biological polymers has demonstrated potential as a remedy for the healing of cutaneous wounds. These polymers shield the wound area from potential contamination, establishing a continuous matrix with the tissue [37].

In conclusion, based on the aforementioned findings, it should be emphasized that the PPPR developed in this study fulfills the fundamental criteria of an effective wound dressing. These criteria include biodegradability, bioavailability, non-toxicity, hydrophilicity, biocompatibility, and ease of handling. Furthermore, the PPPR exhibits favorable antioxidant activity and antibacterial properties and promotes cell migration and proliferation.In addition, it is moisturizing and possesses histocompatibility. When compared to other polymers, the PPPR offers distinct advantages it is cost-effective, easily extracted from readily available natural resources, simple to produce, and can be applied to wounds with ease due to its favorable beneficial properties. The use of chitosan, for example, to accelerate the wound healing process encounters several challenges, including the difficulty of sterilizing chitosan prior to its use in wound care products. Its complex extraction process involves several technical and logistical obstacles [38], similar to other polymers such as alginate and gelatin [39].

## 3. Material and Methods

### 3.1. Chemicals

Alloxan monohydrate butylated hydroxytoluene (BHT), ethylenediaminetetraacetic acid (EDTA), trichloroacetic acid (TCA), potassium ferricyanide, ferric chloride, ketamine, N,O-Bis (trimethylsilyl) trifluoroacetamide (BSTFA), and pyridine were obtained from Sigma-Aldrich. MTT (3-(4,5-dimethylthiazol-2-yl)-2,5-diphenyltetrazoliumbromide) was purchased from Sigma Aldrich (St. Louis, MO, USA).The synthetic reference drug« Cytol Centella^®^ » dermatological cream was purchased from a local pharmacy.

### 3.2. Plant Material

In this study, plant samples of prickly pear (*O. ficus-indica*) cladodes were manually harvested during the month of August 2019 from south Tunisian fields (Sfax city). After removing thorns, fresh cladodes were carefully washed with tap water and cut into approximately 2 cm cubes, then air-dried in the shade at room temperature for 1 month. Dried samples were ground to obtain a fine powder. The obtained flour was then stored in a desiccator until use.

### 3.3. Animals

A total of 20 adult Wistar male rats weighing between 200 and 240 g were kindly provided by the Department of Life Sciences, Faculty of Sciences, at the University of Gabes, Tunisia. All rats were housed in standard polypropylene cages in a controlled environment (22 ± 1 °C; 12 h light/dark cycles) with unrestricted access to water and standard laboratory food.

### 3.4. Extraction and Purification of the PPPR

PPPR extraction was assessed by the method described by Ktari et al. [7]. Briefly, 50 g of prickly pear cladode powder was pre-extracted with 95% ethanol (*v*/*v*) (sample/ethanol, *w*/*v*, 1/20). The residue was then thoroughly extracted with hot distilled water (90 °C) and was later filtered. After the evaporation of filtrates, the concentrated liquid was precipitated by adding ethanol for 24 h at 4 °C and subsequent centrifugation at 4500 rpm for 15 min. The pellet was dissolved in distilled water, and the resulting solution was dialyzed against deionized water. Following dialysis using a membrane with a cut-off of 14 kDa, the solution was frozen at −80 °C and lyophilized, yielding a fine dried powder (PPPR), which was then stored at −20 °C for further studies. Yield was expressed as a percentage (%) of the mass (g) of the PPPR to the mass (g) of prickly pear cladode powder.

### 3.5. Physicochemical Analysis of the PPPR

The ash and moisture contents were estimated according to Association of Official Analytical Chemists (AOAC) standard procedures 930.15 and 942.05, respectively. Fat content was estimated gravimetrically by Soxhlet extraction with hexane. Protein content was determined by the Kjeldahl method. The amount of total sugars was estimated by the phenol-sulfuric acid method. The color of the sample was assessed using a Color Flex spectrocolorimeter (Hunter Associates Laboratory Inc., Reston, VA, USA) and reported as L*, a*, and b* values, which represent of lightness, redness, and yellowness, respectively [40]. All measurements were made in triplicate.

### 3.6. Structural Analysis of the PPPR

#### 3.6.1. Ultraviolet Spectroscopy Detection

The UV absorption spectrum of the PPPR at a final concentration of 0.1% was registered in the wavelength ranging from 200 to 800 nm [40].

#### 3.6.2. Infra-Red Spectroscopic Analysis

The FTIR spectrum of the PPPR was obtained using a Nicolet FTIR spectrometer (Thermo Fisher Scientific, Madison, WI, USA) equipped with a horizontal attenuated total reflection (ATR) accessory. The internal crystal reflector was made from zinc selenide and had a 45° angle of incidence to the IR beam. The spectrum was acquired with a resolution of 4 cm^−1^, and the measurement range was 4000–500 cm^−1^ (mid-IR region) at room temperature. The spectral data were analyzed using the OPUS 3.0 data acquisition software program (Bruker, Ettlingen, Germany).

#### 3.6.3. Monosaccharide Composition Analysis

- TLC and HPLC spectrum

First, 2 mg of PPPR was hydrolyzed in 250 µL of 2 M sulfuric acid (H_2_SO_4_) at 100 °C for 1 h. For HPLC analysis, 20 µL of the hydrolysate was blended with a volume of 980 µL of H_2_O and filtered through a filter with a pore size of 0.45 mm. The monosaccharide composition was analyzed using a Sugar KS-800 column (Showa Denko, Tokyo, Japan) with a mobile phase consisting of 0.001 M NaOH, a flow rate of 0.5 mL/min, and a column temperature of 50 °C. For TLC analysis, sugars were eluted with a mobile phase of chloroform/acetic acid/water in the ratio 6:7:1 (*v*/*v*) on TLC plates (TLC Silica gel 60 F254, (20 × 20 cm), Merck, Saint Quentin Fallavier, France). The TLC plates were stained by spraying with 5% (*v*/*v*) sulfuric acid in ethanol and then visualized after being incubated in the oven at 105 °C for 10 min. Rhamnose, xylose, galactose, glucose, arabinose, mannose, and fructose were used as standard monosaccharides.

- GC–MS analysis

A sample of 20 mg lyophilized hydrolysate was silylated with N,O-Bis (trimethylsilyl) trifluoroacetamide (BSTFA) and pyridine for 1 h at 60 °C. Analysis of the obtained derivatives from the trimethylsilyl sugars was carried out using the Agilent gas chromatography system (Agilent technologies 5977GC/MSD, Santa Clara, CA, USA). The separation and elution conditions were as follows: the injected volume was 1 µL and the temperatures of the detector and the injector were both set at 320 °C. The temperature of the column was set at 100 °C for 1 min and then ramped from 100 to 260 °C at 4 °C/min, and then held for 10 min at 260 °C. The carrier gas used was helium at a flow of 1 mL/min.

#### 3.6.4. Molecular Weight of the PPPR

The molecular weight of the PPPR was determined by a gel filtration chromatography system using a Zorbax PSM 300 column (300 mm × 25 mm) equipped with a refractive index detector. Elution was performed in bi-distilled water at a flow rate of 0.8 mL/min. Dextran was employed as the standard.

### 3.7. DSC Analysis

DSC was employed to characterize the phase transition. Thermal analysis of the PPPR was conducted with a Perkin–Elmer DSC4000 (PerkinElmer, Waltham, MA, USA) equipped with a mechanical cooling system. Six milligrams of PPPR were placed in aluminum [41]. They were then scanned from 0 °C to 350 °C at a rate of 5 °C/min, with an empty aluminum pan used as the reference.

### 3.8. Cytocompatibility (MTT Test)

The cytotoxicity effect of the PPPR on HEK-293 cells was evaluated using the MTT test, as specified by Maalej et al. [42]. Different sample concentrations ranging from 0 to 1600 μg/mL were applied to cells (3 × 10^4^ live cells/mL) for 48 h. The absorbance (A) was then recorded at 570 nm using a microplate reader (Thermo Scientific Varioskan Flash, Waltham, MA, USA). Cells treated with the medium alone were used as the 100% viable control. Blanks for each sample and the control were also prepared under the same conditions.

### 3.9. In Vitro Antioxidant Activities of the PPPR

#### 3.9.1. DPPH Activity

DPPH radical-scavenging activity of the PPPR was carried out as detailed by Ktari et al. [7]. Briefly, a volume of 500 µL of the sample at various concentrations (0.5–10 mg/mL) was mixed with 375 µL of 99.5% ethanol and 125 µL of 0.02% DPPH in 99.5% ethanol. The mixtures were then kept at room temperature in the dark for 60 min, and the reduction of the DPPH radical was detected at 517 nm using a UV–visible spectrophotometer. The outcomes of the radical scavenging via hydrogen donation are clearly visible as a color change from purple to yellow. The control was prepared in the same manner, with distilled water substituted for the sample. Antioxidant activity via DPPH radical scavenging activity was computed by the equation:

DPPH radical-scavenging activity (%) = [(A_control_ − A_sample_)/A_control_] × 100

where A_control_ was the absorbance of the control reaction and A_sample_ was the absorbance of the PPPR. BHT was used as a positive control.

#### 3.9.2. Ferric Reducing Power Assay

The capacity of the PPPR to reduce iron (III) was measured using the method described by Yildirim et al. [43]. A volume of 1 mL of the sample at various concentrations (0.5–10 mg/mL) was added to 2.5 mL of 0.2 M phosphate buffer (pH 6.6) and 2.5 mL of 1% (*w*/*v*) potassium ferricyanide. The mixes were subjected to incubation for 30 min at 50 °C. Following incubation, 2.5 mL of 10% (*w*/*v*) TCA was added to the reaction solutions, which were subsequently centrifuged for 10 min at 10,000 rpm. Finally, 2.5 mL of each sample’s supernatant were combined with 2.5 mL of distilled water and 0.5 mL of ferric chloride (0.1% *w*/*v*). After 10 min, the absorbance of each sample was measured at 700 nm. The control was performed in the same way, with the exception that distilled water was used in place of the sample. BHT served as the antioxidant reference.

#### 3.9.3. Ferrous Chelating Activity Assay

The chelating antioxidant activity of EAP towards ferrous ions (Fe^2+^) was assessed following the protocol explained by Carter [44]. In this method, chelating ability was monitored by measuring the decrease in the intensity of the red color complex (Fe^2+^–ferrozine) at 562 nm. EDTA was utilized as a reference antioxidant compound. The percentage inhibition of ferrozine–Fe^2+^ complex formation was determined using the following equation:

Chelating activity (%) = [(Acontrol − Asample)/Acontrol] × 100

where A_control_ is the absorbance of the control (without the PPPR) and A_sample_ is the absorbance of the reaction tubes (with the PPPR mixed with the ferrozine).

#### 3.9.4. ABTS Assay

The ABTS^+^ radical scavenging activity of the PPPR was evaluated according to the method reported by Braca et al. [45] with minor modifications, across various concentrations ranging from 0.1 to 5 mg/mL. The absorbance of the reaction mixture was spectrophotometrically measured at 734 nm. Trolox served as the standard control. The ABTS^+^ radical scavenging potential was determined using the following equation:

ABTS^+^ radical scavenging activity (%) = [(Acontrol − Asample)/Acontrol] × 100

where A_control_ is the absorbance of the control reaction and A_sample_ is the absorbance of the PPPR.

### 3.10. In Vitro Antibacterial Activity of the PPPR

#### 3.10.1. Determination of Antibacterial Activity with the Disk Diffusion Method

The antibacterial activity of the PPPR was assessed using the agar well-diffusion method and Luria–Bertani (LB) medium. *P. aeruginosa* and *S. Typhimurium* were used as indicator bacteria. The inhibitory effects of the PPPR were tested by examining the growth capacity of the indicator bacteria in the presence of the polysaccharide. Antibacterial activity was assessed by measuring the clear-zone diameter of the bacterial inhibition zone. All tests were performed in triplicate, and the results were averaged. Sterile distilled water served as the negative control.

#### 3.10.2. Determination of the Minimum Inhibitory Concentration (MIC) and Inhibitor Concentration 50% (IC50)

The PPPR’s MIC was assessed against *P. aeruginosa* and *S. Typhimurium*. MIC values represent the lowest concentration needed to completely inhibit the growth of microorganisms according to the MTT assay. Bacterial suspensions were adjusted to 10⁶ CFU/mL to prepare the inoculum for each strain. In a 96-well plate, dilution series of the sample were created, ranging from 40 mg/mL to 0.625 mg/mL. Following a 24 h incubation period at 37 °C, 25 µL of freshly prepared MTT solution (0.5 mg/mL) was added to each well. A change in the medium to a purple color after 30 min indicated that the bacteria were metabolically active. All experiments were conducted in duplicate, and the IC50 is the concentration of the compound required to reduce bacterial activity by 50% compared to the negative control consisting of cultures in LB medium without additional PPPR. The MIC was defined as the lowest concentration at which the MTT color change was not observed. Cultures grown in LB medium without additional PPPR were treated as the control.

### 3.11. In Vivo Wound Healing Potential of the PPPR in Diabetic Rats

The PPPR was dissolved in glycerol solution to a final concentration of 15 mg/mL and the mixture was agitated until a hydrogel was formed.

#### 3.11.1. Induction Type1 Diabetes Mellitus, Full-Thickness Skin Wound Creation, and Experiment Protocol

All experiments on rats were conducted according to the Guide for Care and Use of Laboratory Animals provided by the University of Sfax, Tunisia, and were approved by the Animal Ethics Committee. To prevent animals from licking or biting each other’s wounds, they were housed in individual cages.

After acclimatization for one week (temperature (22–25 °C), relative humidity (60–70%), and a 12 h dark/12 h light cycle), the animals were injected intraperitoneally with a freshly prepared solution of alloxan monohydrate dissolved in physiological saline solution, at a dose of approximately120 mg/kg body weight. To prevent severe hypoglycemia, rats were given a 20% glucose solution after 6 h, followed by a 5% glucose solution for the next 24 h. The diagnosis of type1 diabetes mellitus was confirmed 3 days after induction using a glucometer through the tail vein. The rats with hyperglycemia of more than 2 g/L were chosen for the experiment. Every three days, the animal’s glycemia was checked to ensure it maintained high blood glucose levels. After 15 days, mechanical wound induction in diabetic Wistar rats was performed as previously reported by Chouikhi et al. [46]. Briefly, twenty diabetic rats were first totally anesthetized via intraperitoneal injection of 50 mg/kg ketamine, then round dorsal surfaces of the skin were shaved with an electronic shaver. Finally, full-thickness round wounds of approximately 1.5–2 cm^2^ were applied to the shaved region of all diabetic rats using surgical scissors. To prevent communal licking, rats were separated into individual cages.

All rats were randomly divided into four groups of five animals each: Group 1 consisted of rats treated with physiological serum and was designated as the control group. Group 2 comprised rats treated with “CytolCentella^®^”, designated as the reference group. Group 3 consisted of glycerol-treated rats, while Group 4 comprised PPPR-treated rats. After rinsing wounds with physiological serum, the standard drug “Cytol Centella^®^”, glycerol, and the PPPR were applied to the surface of the wound in a thin layer every two days until the wound had completely healed. After each wound treatment application, wounds were covered with compression dressings. The experiment began on the wound induction day (day 1) and continued until the first group had totally healed (day 14). On the final day, animals anesthetized with ether were sacrificed by decapitation and the granulation tissues were removed. A sample of moist tissue was promptly preserved in a 10% buffered formalin solution for further histological observations.

#### 3.11.2. Macroscopic Analysis

To assess wound healing efficiency, wounds were photographed on days 1, 3, 5, 10, 12, and 14 post-wounding using a panasonic LUMIX digital camera (LUMIX FZ1000 4K QFHD/HD 16X, Wiesbaden, Germany) and colorimetric analysis was conducted. In parallel, decreases in the wound areas were monitored using the Autodesk Auto CAD 2015 software application for designing and drafting [46] and then translated into percent values, with the wound size at the time of wounding set to 100%, using the following equation:

$$Wound\;closure\;rate=\frac{(wound\;size\;\left(day\;1\right)-wound\;size\;\left(day\;n\right)}{Wound\;size\;\left(day\;1\right)}\times 100$$

#### 3.11.3. Histological Examinations

Tissue samples were taken from the wound site of all the studied groups. They were explanted and fixed in 10% neutral-buffered formalin, and then embedded in paraffin. The sections were then stained with hematoxylin–eosin and examined under a light microscope (×400 magnification) to assess collagen production, fibroblast proliferation, epithelialization, and angiogenesis.

### 3.12. Statistical Analysis

All data are presented as mean ± standard deviation (SD) of three values and were processed using SPSS ver 22.0. Differences were considered statistically significant at *p <* 0.05.

## 4. Conclusions

In this work, a chain of glucose, mannose, galactose, and rhamnose was isolated as a heteropolysaccharide from Prickly pear (*O. ficus-indica*) rackets. Monosaccharide composition analysis of the PPPR revealed that glucose is the most prevalent monosaccharide present. In fact, glucose represents 62.4%, galactose 19.37%, mannose 10.24%, and rhamnose 7.98%, providing valuable insights into the polysaccharide’s structure and potential properties. This novel polysaccharide had an average molecular weight of 90.94 kDa and possessed promising antioxidant potential, particularly to scavenge DPPH radicals and lower levels of ferric ions. Moreover, the PPPR exhibited notable antibacterial activities against *P. aeruginosa* and *S. Typhimurium.* Furthermore, the significant improvement in wound healing provided by the PPPR in an alloxan-induced diabetic rat model highlights its potential as an effective treatment for wound management. This suggests that the PPPR could offer substantial benefits in promoting faster and more effective wound recovery. Additionally, this study might provide a fresh approach to using Tunisian rackets from Prickly pear (*O. ficus-indica*) as an important bioresource for the large-scale synthesis of biomaterials with added value, which would undoubtedly expand their growth and application.

## Figures and Tables

**Table 1:** Physicochemical properties of the PPPR.

Parameters	PPPR
Yield (%)	23.17 *±* 0.36
Moisture (%)	5.11 ± 0.25
Polysaccharides (%)	91.02 ± 0.6
Proteins (%)	1.1 ± 0.01
Fat (%)	0.00
Ash (%)	2.69 ± 0.54
Color	
L*	92.44 ± 0.05
a*	0.17 ± 0.00
b*	2.23 ± 0.11

Physicochemical composition was calculated based on the dry matter. Values are given as mean ± SD from triplicate determinations (n = 3).

**Table 2:** Results of GC–MS analysis of the PPPR.

Peak	RT	Area (%)	Compounds
1	14.529	2.09	L-Rhamnose, (R,R,S,S)-, 4TMS derivative
2	14.774	5.89	D-Rhamnopyranose, 4TMS derivative (isomer 1)
3	16.492	10.24	α-L-Mannofuranose, 6-deoxy-1,2,3,5-tetrakis-O-(trimethylsilyl)-
4	17.018	6.06	Methyl α-D-glucofuranoside, 4TMS derivative
5	17.13	8.14	D-Glucopyranose, 5TMS derivative
6	17.5	27.09	D-Glucose, 5TMS derivative
7	17.609	20.81	β-D-Glucopyranose, 5TMS derivative
8	18.331	19.37	D-(+)-Galactopyranose, 5TMS derivative (isomer 2)

## Data Availability

No new data were created or analyzed in this study.
